# A survey on Helicobacter pylori infection rate in Hainan Province and analysis of related risk factors

**DOI:** 10.1186/s12876-023-02973-3

**Published:** 2023-09-30

**Authors:** Run-xiang Chen, Da-ya Zhang, Xiaodong Zhang, Shiju Chen, Shimei Huang, Chen Chen, Da Li, Fan Zeng, Jiajia Chen, Cuiyi Mo, Lei Gao, Juntao Zeng, Jianxin Xiong, Zhai Chen, Feihu Bai

**Affiliations:** 1 Graduate School, Hainan Medical University, Haikou, 571199 China; 2 Department of Gastroenterology, Qionghai People’s Hospital, Qionghai, 571400 China; 3 Department of Gastroenterology, Sanya Central Hospital, Sanya, 572022 China; 4 Department of Gastroenterology, Hainan Second People’s Hospital, Wuzhishan, 572299 China; 5 Department of Gastroenterology, Dongfang People’s Hospital, Dongfang, 572699 China; 6 Department of Gastroenterology, The Second Affiliated Hospital of Hainan Medical University, Yehai Avenue, #368, Longhua District, Haikou, 570216 Hainan Province China; 7 The Gastroenterology Clinical Medical Center of Hainan Province, Haikou, 570216 China

**Keywords:** Hainan, *H. pylori*, Prevalence, Risk factor

## Abstract

**Objective:**

The aim of this study was to understand the prevalence and potential risk factors of *Helicobacter pylori* (*H. pylori*) infection in Hainan Province, China.

**Methods:**

We conducted this study in 21 health service stations in 5 cities of Hainan Province from August 2022 to April 2023. We selected the various participants based on a stratified whole-group sampling method. The 14C-UBT was used to analyze *H. pylori* infection in 3632 participants. We also analyzed the possible relationship between variables and *H. pylori* infection based on chi-square test and multifactorial logistic regression. The model was evaluated by performing a Hosmer–Lemeshow goodness-of-fit test and plotting receiver operating characteristic(ROC) curves.

**Results:**

In total, the results of 3632 eligible participants (age: 14 to 93 years) were included in the analysis. The total prevalence of *H. pylori* infection in Hainan Province was approximately 38.7%. The prevalence of *H. pylori* infection was found to increase with age, stabilized in the age group of 45 to 64 years, but peaked in the age group of 65 years and older. In multifactorial analysis, the prevalence of *H. pylori* infection was positively associated with middle-aged adults (45–64 years), older adults (≥ 65 years), drinking, farmers, natural labor, routinely share utensils, have habit of frequent betel nut consumption, upper gastrointestinal symptoms, and family history of gastric cancer. The factors negatively associated with prevalence included family size ≤ 3, washing hands often before meals, frequent exercise, regular meals, and frequent consumption of fruits and vegetables. In addition, the Hosmer–Lemeshow test showed a good fit (χ^2^ = 12.983, *P* = 0.112) and the area under ROC was 0.631 (95%CI: 0.613 ~ 0.649).

**Conclusion:**

The prevalence of *H. pylori* infection in Hainan Province was observed to be moderate and closely related to age, local socioeconomic conditions, hygienic status and dietary habits.

## Introduction

*Helicobacter pylori* (*H. pylori*) is a Gram-negative bacillus, hat primarily colonizes in the gastric epithelium and was first discovered in 1983 by Marshall and Warren, for which they were awarded the Nobel Prize in Medicine [1]. *H. pylori* has been identified as a major cause of gastritis, peptic ulcers, as well as gastric cancer, and is the only bacterium classified as a class I carcinogen by the International Agency for Research on Cancer (IARC) [2, 3]. In addition, it has also been closely associated with several extra-gastrointestinal diseases [4].

*H. pylori* infects approximately half of the global population, but the prevalence varies widely between different countries and regions [5]. Although the prevalence is decreasing rapidly in most developed countries, it remains significantly high in most developing countries [6]. Epidemiological studies have revealed that the prevalence of *H. pylori* infection in China ranges from 28%-82% [5], and its overall prevalence is approximately 44.2% [7]. Accumulating evidences have suggested that different factors such as gender, age, occupation, education, socioeconomic status, diet, lifestyle, and number of family members can influence the prevalence of *H. pylori* in the population [8–10]. Age is an important variable that has been the focus of most published studies, but the age group associated with the highest prevalence has not yet reached a consensus. Additionally, some studies have reported that *H. pylori* infection can be acquired mainly in early childhood [11–13], whereas others have shown that the prevalence of infection increases significantly with age [14–16]. However, the potential correlation between these factors and *H. pylori* infection is inconclusive.

The relevant data on the prevalence of *H. pylori* in the southern coastal provinces of china are still lacking. Therefore, our aim was to determine the prevalence of *H. pylori* infection in Hainan Province and to assess the different risk factors associated with *H. pylori* infection based on the data collected from various regions of Hainan Province.

## Materials and methods

### Study population and design

We conducted this study from August 2022 to April 2023 in Hainan Province, China, which included 21 different health service stations from 5 cities (Haikou, Sanya, Qionghai, Dongfang, and Wuzhishan). Inclusion criteria: (1) each participant included in this study was 14 years old and above who were able to complete 14C-UBT and questionnaire; (2) each participant is a native of Hainan or has at least 10 years of residence history. Exclusion criteria included treatment for *H. pylori* within the past 3 months, antibiotic use within 1 month, and use of proton pump inhibitors and bismuth agents within 2 weeks. In addition, other exclusion criteria taken into consideration were severe cardiac, hepatic or renal insufficiency and contraindications to 14C-UBT. The objectives of this study were explained in detail to all participants, and also, partici-pating in this study was fully conscious and based on their desire. Further, written informed consent was obtained from each participant. Meanwhile, each participant provided a written or electronic informed consent form and the study was approved by the institutional ethics committee of the Second Hospital of Hainan Medical University (reference number: LW20221025).

### Questionnaire and definition

The questionnaire from the previous studies in China was referred to and modified accordingly [8–10]. The content of the questionnaire mainly included questions related to the demographic characteristics (e.g., age, gender, ethnicity, occupation, marital status, and BMI), socioeconomic status (e.g., education level and annual personal income), hygiene habits (e.g., sharing utensils, absorption before meals, washing hands after the stools, frequency of brushing teeth), lifestyle (e.g., smoking, exercise, work stress, sleep status), dietary habits (eating out, drinking water, alcohol, tea, fruit and vegetable consumption, etc.) and family history of gastric cancer. The data was entered by two different individuals and double-checked to minimize errors. Gastrointestinal symptoms include nausea, vomiting, acid reflux, heartburn, abdominal pain, bloating, constipation and more. Smoking was defined as smoking at least one cigarette per day or having smoked in the past 1 year. Alcohol consumption was defined as consuming at least 100 g of alcohol per week in the past 1 year. "Regular" was defined as at least once a day, and "occasional" was defined as once every 2–3 days or more. Body Mass Index (BMI) is a person’s weight in kilograms (or pounds) divided by the square of height in meters (or feet). Based on BMI, < 18.5 was the low-weight group, ≥ 18.5 and < 24 were the normal weight group, ≥ 24 were overweight group or obese group.

### Carbon-14 urea breath test (14C-UBT)

The subjects received 14C breath test in the morning on an empty stomach or fasted for more than 2 h. The test method was strictly in accordance with the instructions. The test was performed by swallowing a 14C urea capsule with an appropriate amount of pure water and then sitting for 15–20 min. It was followed by blowing into the breath card for about 3–5 min, and sampling was completed when the color of the indicator tablet changed from orange to yellow to indicate the presence of *H. pylori* infection.

### Sample size calculation

This study focused on the prevalence of *H. pylori* infection as the main study index, and the overall prevalence in China is known to be approximately 44.2% based on the previously published literature [7]. Thus, by using the following formula, α = 0.05 (two-sided test) was selected, with an allowable error δ =  ± 1.7%, Z_(1-α/2) = 1.96, and *p* = 44.2%. The calculation yielded the need to include 3279 cases. However, considering the shedding rate of 10%-20%, a total of 3665 cases were included in the study, which can ensure both the accuracy as well as scientific validity of the study results.

### Statistical analysis

SPSS 25.0 software was used to analyze the data. The continuous variables were expressed as mean ± standard deviation (SD), and independent sample t-test was used for comparison between the groups. The categorical variables were expressed as frequencies and percentages [n(%)], and comparisons between the groups were made using chi-square tests or trend chi-square tests. All the variables with p < 0.05 in the univariate analysis were included in the multifactorial logistic regression analysis (stepwise regression analysis, SLS = 0.10, SLE = 0.05) to explore the potential relationship between *H. pylori* infection and risk factors. The results were expressed as the dominance ratio (OR) with a confidence interval (CI) of 95%. The model was evaluated by performing a Hosmer–Lemeshow goodness-of-fit test and plotting receiver operating characteristic (ROC) curves. The differences were considered statistically significant when the *P* value was less than 0.05.

## Result

### Demographic characteristics of the investigated population

**Fig. 1:**
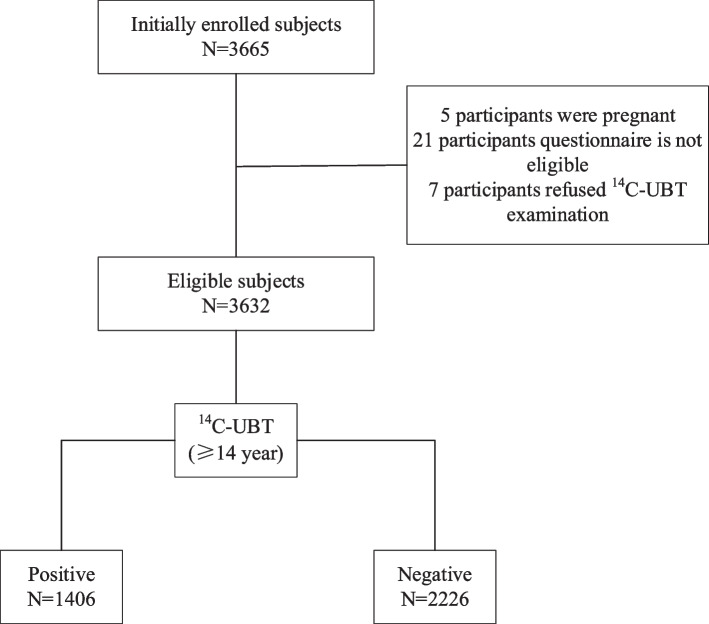
Study flowchart (n, number;14C-UBT, carbon-14 urea breath test)

A total of 3665 subjects were initially recruited in this study, some of whom were excluded due to ineligible questionnaires or unwillingness to undergo the 14C urea breath test. Ultimately, 3632 eligible subjects were included in the final analysis. Among these, 1356 (37.3%) were male and 2276 (62.7%) were female, with an age range of 14–93 years and a mean age of 48.0 ± 14.8 years (Fig. 1).

### *H. pylori* infection rate in the Hainan province

**Fig. 2:**
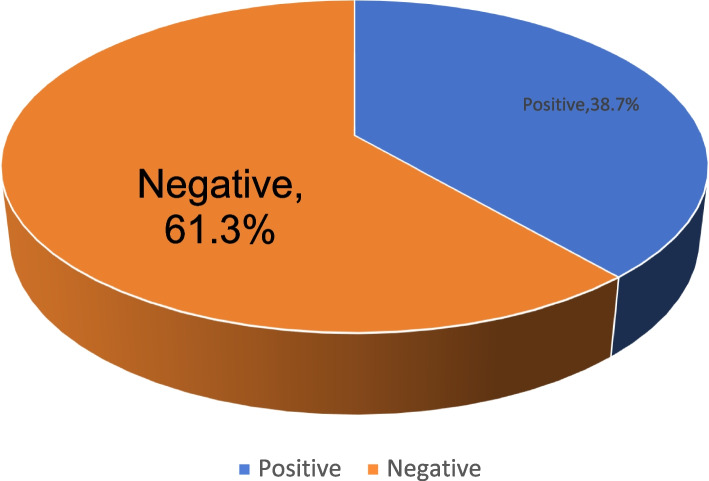
Overall positivity rate of *H. pylori* in Hainan Province

**Fig. 3:**
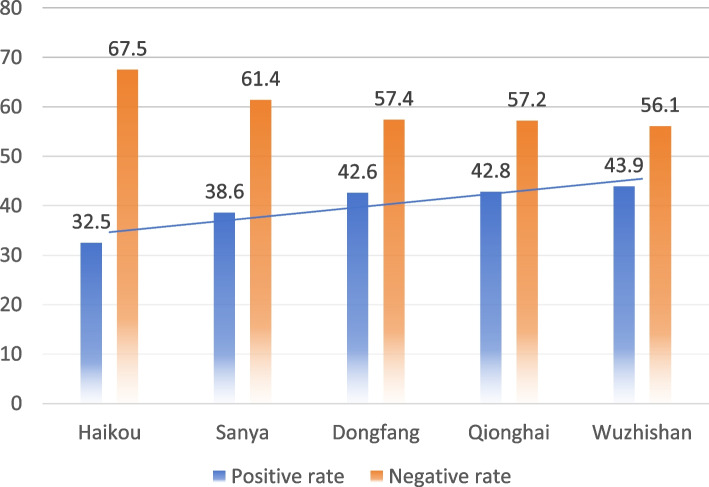
Prevalence of *H. pylori* in various regions of Hainan Province

The total infection rate of *H. pylori* in Hainan Province was found to be 38.7% (Fig. 2). The prevalence of infection in each city and county, in descending order, was Wuzhishan (43.9%), Qionghai (42.8%), Dongfang (42.6%), Sanya (38.6%), and Haikou (32.5%) respectively with statistically significant differences (*P* < 0.001) (Fig. 3). The prevalence of *H. pylori* infection was observed to increase with age, with the highest rate of *H. pylori* infection in people over 65 years old (43.4%) and the lowest rate in people under 18 years old (20.7%), with a statistically significant difference (*P* < 0.001). The prevalence of infection was slightly higher in women (39.9%) in comparison to men (36.8%), but the difference was not statistically significant (*P* = 0.068) (Table 1).

**Table 1:** Univariate analysis of factors associated with *H. pylori* infection [cases (%)]

Variables	Positive	Negative	χ^2^	*P*-value
Age(years)				
< 18	12 (20.7)	46 (79.3)	25.739	< 0.001
18 ~	521 (35.0)	966 (65.0)		
45 ~	622 (41.2)	886 (58.8)		
> 65	251 (43.4)	328 (56.6)		
Gender				
Male	499 (36.8)	857 (63.2)	3.334	0.068
Female	907 (39.9)	1369 (60.1)		
Ethnicity				
Han	966 (39.7)	1466 (60.3)	3.158	0.076
Li	440 (36.7)	760 (63.3)		
Occupation				
Farmer	474 (41.4)	670 (58.6)	5.216	0.022
Non-farmer	932 (37.5)	1556 (62.5)		
Previous infections				
Yes	263 (36.2)	464 (63.8)	2.463	0.117
No	1143 (39.3)	1762 (60.7)		
Same bed				
Yes	1030 (40.5)	1515 (59.5)	11.103	0.001
No	376 (34.6)	711 (65.4)		
Family size				
≤ 3	492 (30.8)	1108 (69.3)	76.403	< 0.001
> 3	914 (45.0)	1118 (55.0)		
Family infection				
Yes	267 (40.2)	397 (59.8)	0.770	0.380
No	1139 (38.4)	1829 (61.6)		
Delivery method				
Natural labor	1298 (39.4)	1995 (60.6)	7.401	0.007
Cesarean section	108 (31.9)	231 (68.1)		
Breast feeding				
Yes	1255 (38.3)	2018 (61.7)	1.884	0.170
No	151 (42.1)	208 (57.9)		
Body mass index(BMI, kg/m^2^)				
< 18.5	89 (41.2)	127 (58.8)	2.048	0.359
18.5 ~	447 (40.1)	669 (59.9)		
> 24.0	869 (37.9)	1424 (62.1)		
Education level				
Primary education	739 (43.1)	977 (56.9)	33.547	< 0.001
Secondary education	352 (38.0)	575 (62.0)		
Higher education	1406 (31.9)	2226 (68.1)		
Personal monthly income				
< 3 k	870 (40.8)	1260 (59.2)	10.667	0.005
3 k-5 k	401 (36.4)	702 (63.6)		
> 5 k	135 (33.8)	264 (66.2)		
Frequency of sharing utensils with others				
Frequent	1015 (41.0)	1461 (59.0)	17.076	< 0.001
Never or occasionally	391 (33.8)	765 (66.2)		
Washing hands before meals				
Frequent	932 (35.9)	1662 (64.1)	29.617	< 0.001
Never or occasionally	474 (45.7)	564 (54.3)		
Washing hands after using the toilet				
Frequent	1222 (37.9)	2004 (62.1)	8.414	0.004
Never or occasionally	184 (45.3)	222 (54.7)		
Brushing(frequency/day)				
None or 1 time	579 (42.8)	775 (57.2)	14.930	< 0.001
2 time and above	827 (36.3)	1451 (63.7)		
Smoking				
Yes	194 (36.7)	335 (63.3)	1.084	0.298
No	1212 (39.1)	1891 (60.9)		
Frequent exercise				
Yes	890 (35.0)	1650 (65.0)	48.013	< 0.001
No	516 (47.3)	576 (52.7)		
Work stress				
Frequent	790 (37.4)	1324 (62.6)	3.837	0.05
Never or occasionally	616 (40.6)	902 (59.4)		
Sleeping				
Good	1141 (39.4)	1756 (60.6)	2.742	0.098
Weak	265 (36.1)	470 (63.9)		
Dining out				
Frequent	300 (40.4)	443 (59.6)	1.092	0.296
Never or occasionally	1106 (38.3)	1783 (61.7)		
Types of drinking water				
Boiling tap water	1151 (38.8)	1816 (61.2)	0.396	0.820
Bottled mineral water	173 (39.1)	269 (60.9)		
Well water	82 (36.8)	141 (63.2)		
Regular meals				
Yes	1071 (36.2)	1888 (63.8)	42.634	< 0.001
No	335 (49.8)	338 (50.2)		
Drinking				
Yes	419 (41.4)	592 (58.6)	4.409	0.036
No	987 (37.7)	1634 (62.3)		
Frequency of drinking tea				
Frequent	981 (39.3)	1517 (60.7)	1.057	0.304
Never or occasionally	425 (37.5)	709 (62.5)		
Frequency of fresh vegetable and fruit consumption				
Frequent	1020 (36.1)	1803 (63.9)	35.548	< 0.001
Never or occasionally	386 (47.7)	423 (52.3)		
Frequency of pickle consumption				
Yes	301 (41.6)	422 (58.4)	3.245	0.072
No	1105 (38.0)	1804 (62.0)		
Frequency of betel nut consumption				
Frequent	139 (47.9)	151 (52.1)	11.291	0.001
Never or occasionally	1267 (37.9)	2075 (62.1)		
Family history of gastric cancer				
Yes	104 (61.9)	64 (38.1)	39.938	< 0.001
No	1302 (37.6)	2162 (62.4)		
Upper gastrointestinal symptoms				
Yes	649 (46.1)	759 (53.9)	52.818	< 0.001
No	757 (34.0)	1467 (66.0)		

### Association between variables and *H. pylori* infection

Univariate analysis revealed that *H. pylori* prevalence was significantly associated with age, occupation, education, same bed, family size, personal monthly income, frequency of sharing utensils with others, brushing, washing hands before meals, washing hands after using the toilet, mode of delivery, family history of gastric cancer, dietary pattern, frequent exercise, alcohol consumption, frequency of eating fruits and vegetables, frequency of eating betel nut, and gastrointestinal symptoms. In addition, other variables were not significantly associated with *H. pylori* infection (Table 1).

### Factors independently associated with *H. pylori* infection and prediction model

**Fig. 4:**
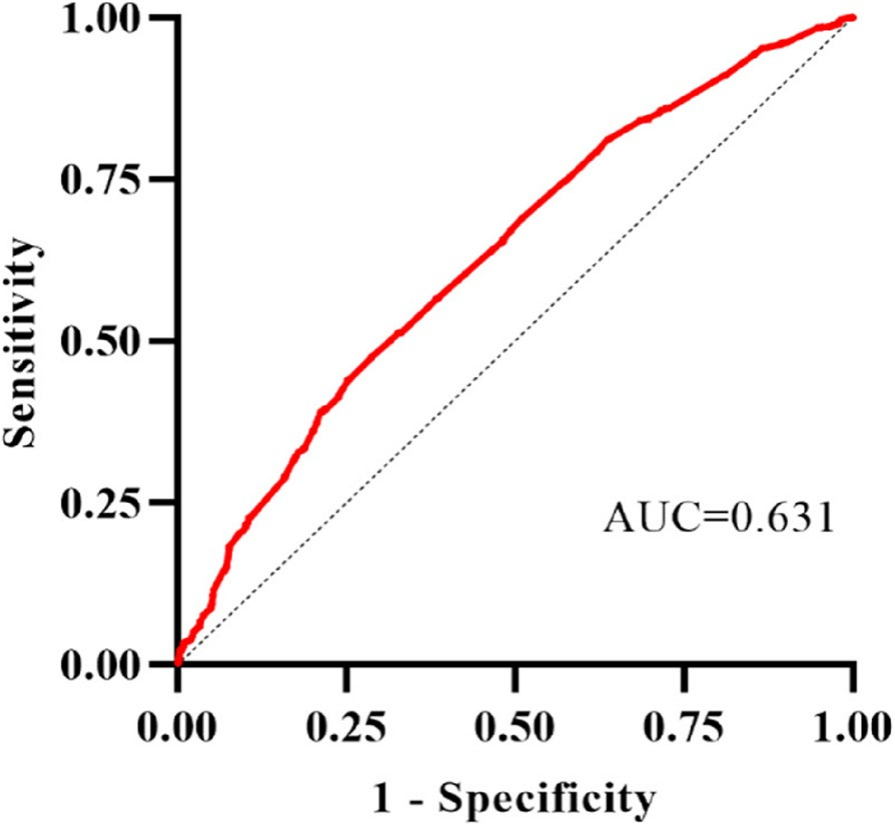
Receiver operating characteristic curve of prediction model for *H. pylori* infection

These significant variables were further assessed by including them in a multifactorial logistic regression model, as depicted in Table 2. Middle-aged adults (45–64 years) (odds ratio [OR]:2.154; 95% confidence interval [CI]:1.100–4.218), older adults (≥ 65 years) (OR:2.181, 95% CI:1.097–4.339), farmers (OR:1.266, 95% CI:1.062–1.509), natural labor (OR:3.622, 95% CI:2.544–5.156), frequently share utensils (OR:1.241, 95% CI:1.230–1.685), drinking (OR:1.385, 95% CI: 1.169–1.640), frequent betel nut consumption (OR:1.306, 95% CI:1.005–1.697), upper gastrointestinal symptoms (OR:1.501, 95% CI:1.289–1.747), and family history of gastric cancer (OR: 3.807, 95% CI: 2.666–5.438) were found to be positively associated with *H. pylori* infection. In addition, the factors negatively associated with prevalence included family size ≤ 3 (OR:0.695, 95% CI:0.594–0.813), washing hands often before meals (OR:0.775, 95% CI:0.638–0.941), frequent exercise (OR:0.565. 95% CI:0.479–0.667), regular meals (OR:0.481, 95% CI:0.384–0.603), and frequent consumption of fruits and vegetables (OR:0.601, 95% CI:0.529–0.752) (Table 2). In addition, the Hosmer–Lemeshow test showed a good fit (χ^2^ = 12.983, *P* = 0.112) and the area under ROC was 0.631 (95%CI: 0.613 ~ 0.649) (Fig. 4).

**Table 2:** Multivariate logistic regression analysis of *H. pylori* infection in the 3632 subjects above 14 years old

Variables	*OR*	95%*CI*	*P-value*
Age			
< 18	Reference		
18 ~	1.750	0.889–3.445	0.106
45 ~	2.154	1.100–4.218	0.025
> 65	2.181	1.097–4.339	0.026
Farmer	1.266	1.062–1.509	0.009
Natural labor	3.622	2.544–5.156	< 0.001
Family size ≤ 3	0.695	0.594–0.813	< 0.001
Frequently share utensils	1.241	1.230–1.685	< 0.001
Washing hands often before meals	0.775	0.638–0.941	0.010
Frequent exercise	0.565	0.479–0.667	< 0.001
Regular meals	0.481	0.384–0.603	< 0.001
Drinking	1.385	1.169–1.640	< 0.001
Regular consumption of fruits and vegetables	0.601	0.529–0.752	< 0.001
Frequent betel nut consumption	1.306	1.005–1.697	0.046
Upper gastrointestinal symptoms	1.501	1.289–1.747	< 0.001
Family history of gastric cancer	3.807	2.666–5.438	< 0.001

## Discussion

This study was the first to investigate the prevalence of *H. pylori* and its associated risk factors in Hainan Province, China. We used 14C-UBT to detect *H. pylori* infection in people aged 14 years or older. The results indicated that the overall prevalence of *H. pylori* infection in Hainan Province was 38.7%, which was significantly lower than the overall prevalence of 44.2% in China (95% CI: 43.0–45.5%) and in line with the global trend of a continuous decrease in *H. pylori* infection in recent years [6, 7]. This is inextricably linked to China's high economic growth, high level of universal health coverage, and improved public health conditions [17]. However, we also noted that only 9.7% (355/3632) of the population had previously received *H. pylori* screening and eradication treatment. This observation suggests that good scientific knowledge of *H. pylori* can potentially help to improve the awareness of *H. pylori* and alleviate the existing situation of *H. pylori* infection.

The prevalence of *H. pylori* infection was found to increase sharply with age but plateaus in the middle-aged and older age groups above 45 years. This result was similar to several large-scale epidemiological surveys in China [9, 10]. There could be two possible reasons to explain these findings. On the one hand, the rapid economic and health development in China in the last two decades has resulted in relatively few risk factors associated with *H. pylori* exposure in young people, which has markedly reduced the risk of infection. On the other hand, middle-aged and elderly people have a relatively less stable gastric environment as well as weaker organism resistance, and all of these factors can favor the colonization of *H. pylori*. However, there is no conclusive evidence regarding the possible relationship between gender and *H. pylori* infection. For instance, in one study Moshkowitz et al. suggested that *H. pylori* were more likely to colonize the gastric mucosa in women [18], but our study did not find a significant correlation between the gender and *H. pylori* infection, and this finding was in agreement with conclusions of several previous studies [8, 9].

Occupation is an important factor influencing *H. pylori* infection, which was consistent with the results of previous studies [8–19]. It is generally believed that occupational groups working in agriculture have a relatively higher risk of *H. pylori* infection, and multiple reasons may be involved to explain this finding. First, farmers generally have a low level of education and often have poor hygiene habits and lifestyles; and second these individuals often have a low socioeconomic status as well as limited access to medical resources for *H. pylori* screening and eradication treatment. Moreover, we found regional differences in the prevalence of *H. pylori* infection in Hainan Province, with economically developed areas (e.g., Haikou City) having significantly higher rates of *H. pylori* infection in comparison to economically backward areas (e.g.,Wuzhishan City). This observation also indirectly indicated that socioeconomic status was closely related to *H. pylori* infection.

It is now recognized that oral-oral, fecal–oral is the main transmission route of *H. pylori* [20]. In China, sharing of meals is an important food culture, but this can effectively increase the risk of *H. pylori* transmission and infection [14]. This was confirmed in the present study as it was found that people who share tableware are more likely to be infected with *H. pylori*. In addition, poor hygiene practices can substantially increase the risk of *H. pylori* transmission and serve as an important factor influencing *H. pylori* infection [21]. In our study, frequent premeal hand washing was identified as independent protective factors for *H. pylori* infection. Therefore, introduction of meal sharing and the promotion of personal hygiene as well as health awareness could be useful public health measures for the prevention of *H. pylori* infection.

A good lifestyle seems to be effective in reducing the risk of *H. pylori* infection and gastric cancer development [22]. We found that the prevalence of *H. pylori* infection was significantly lower in participants who participated in regular exercise, had regular meals and maintained a regular diet of fruits and vegetables compared to other populations who did not had such healthy habits, and they were also independent protective factors for *H. pylori* infection. The possible reasons could be as following (i) the presence of chemicals such as isothiocyanate radicicicin and mustard oleoresin in fruits and vegetables can effectively inhibit the colonization of *H. pylori* and thereby reduce the inflammatory response of the gastric mucosa [23]. (ii) Regular exercise can enhance the body's immunity and regular diet can contribute to the stability of the gastric environment, all of which can significantly reduce the risk of *H. pylori* colonization. In our study, regular betel nut as well as alcohol consumption were associated with increased odds of *H. pylori* infection. The practice of washing the raw leaves of betel vine and betel nut (used in the preparation of betel nut liquid) in unclean water may explain our findings [24]. We also found that alcohol consumption is an independent risk factor for *H. pylori* infection. It is well established that home-brewing is more common in Hainan Province, but the production process is crude and the relevant indicators remain untested. A number of previous studies have reported that alcohol consumption has a protective effect or is not associated with *H. pylori* infection, probably due to increased gastric acid secretion and promotion of gastric emptying after alcohol intake, as alcohol has a powerful and direct antibacterial activity, thus potentially inhibiting the growth of *H. pylori* in the stomach. In addition, alcohol can promote gastric emptying and reduce the contact time between *H. pylori* and the gastric mucosa, which in turn can attenuate the rate of *H. pylori* infection [25, 26]. In particular, red wine was found to prevent *H. pylori*-induced gastritis in a mouse model [27]. Another study [28] reported alcohol as a significant risk factor for *H. pylori* infection because alcohol can directly damage the gastric mucosal layer, thus weakening the gastric mucosal defense barrier. Thus, alcohol could theoretically provide a pathway for *H. pylori* infection, while heavy alcohol consumption may predispose consumers to different social contacts that favor the transmission of *H. pylori* infection. Therefore, the potential relationship between alcohol consumption and *H. pylori* infection still requires further longitudinal and epidemiological studies for further explanation.

*H. pylori* infection serves as an important influencing factor in the development of gastric cancer. For instance, a large cohort study found that the incidence of gastric cancer increased each year with the duration of *H. pylori* infection [29]. In addition, the risk of gastric cancer as well as the risk of death decreased significantly after *H. pylori* eradication [30]. Similar to a previous study, we found that a family history of gastric cancer can serve as an independent risk factor for *H. pylori* infection [31]. Upper gastrointestinal symptoms were identified as one of the important risk factors for *H. pylori* infection in this study. However, asymptomatic infection is an important feature of most *H. pylori*-infected patients, who often have developed severe upper GI disease by the time they seek medical attention. Therefore, one should consider upper GI symptoms (e.g., nausea, acid reflux) as an early warning sign of *H. pylori* infection, which can be an important step for the timely diagnosis and treatment of *H. pylori*.

The strengths of this study include the fact that this was the first large-scale epidemiological investigation of *H. pylori* in Hainan Province, China. In addition, the method employed to detect *H. pylori* infection was highly accurate and specific. Of course, the study has some limitations. First, cross-sectional studies do not accurately reflect the temporal trends in *H. pylori* infection. Second, outcome and exposure were assessed simultaneously, which does not allow for a temporal relationship to be established. Finally, the age of our subjects was limited to those aged 14 years or older, thus failing to cover the entire age range in Hainan Province.

In conclusion, the prevalence of *H. pylori* infection in Hainan Province was significantly lower than the overall prevalence in China and closely related to age, local socioeconomic conditions, hygienic status and dietary habits.

## Data Availability

The datasets generated and/or analyzed during the current study are available from the corresponding author upon reasonable request.

## Abbreviations

*H. pylori*: Helicobacter pylori
^14^C-UBT: Carbon-14 urea breath test
SD: Standard deviation
CI: Confidence interval
ORs: Odds ratios
